# Current and Emerging Technologies for the Detection of Norovirus from Shellfish

**DOI:** 10.3390/foods8060187

**Published:** 2019-05-31

**Authors:** Pradip Gyawali, Sanjaya KC, David J. Beale, Joanne Hewitt

**Affiliations:** 1 Institute of Environmental Science and Research Ltd. (ESR), Porirua 5240, New Zealand; joanne.hewitt@esr.cri.nz; 2 Institute for Molecular Bioscience, The University of Queensland, Brisbane, QLD 4072, Australia; sanjaya.kc@imb.uq.edu.au; 3 Commonwealth Scientific and Industrial Research Organization, Ecoscience Precinct, Dutton Park, QLD 4102, Australia; david.beale@csiro.au

**Keywords:** norovirus in shellfish, detection methods, emerging technologies, sensing norovirus

## Abstract

Reports of norovirus infections associated with the consumption of contaminated bivalve molluscan shellfish negatively impact both consumers and commercial shellfish operators. Current virus recovery and PCR detection methods can be expensive and time consuming. Due to the lack of rapid, user-friendly and onsite/infield methods, it has been difficult to establish an effective virus monitoring regime that is able to identify contamination points across the production line (i.e., farm-to-plate) to ensure shellfish quality. The focus of this review is to evaluate current norovirus detection methods and discuss emerging approaches. Recent advances in omics-based detection approaches have the potential to identify novel biomarkers that can be incorporated into rapid detection kits for onsite use. Furthermore, some omics techniques have the potential to simultaneously detect multiple enteric viruses that cause human disease. Other emerging technologies discussed include microfluidic, aptamer and biosensor-based detection methods developed to detect norovirus with high sensitivity from a simple matrix. Many of these approaches have the potential to be developed as user-friendly onsite detection kits with minimal costs. However, more collaborative efforts on research and development will be required to commercialize such products. Once developed, these emerging technologies could provide a way forward that minimizes public health risks associated with shellfish consumption.

## 1. Introduction

Human fecal contamination from sewage discharge, septic tank leaks/overflows, recreational activities and storm water runoff are major sources of norovirus contamination in coastal waters (i.e., shellfish growing areas) [1]. While environmental (temperature, salinity), physical (dilution, turbidity, transportation) and biological (inactivation) factors can reduce norovirus concentrations in shellfish-growing waters, efficient bioaccumulation in shellfish can result in levels of contamination that pose a risk to consumers as norovirus has a low (~18 viral particles) infectious dose [2].

Norovirus in shellfish can be difficult to remove or inactivate using simple post-harvest treatments such as depuration and relaying [3,4,5]. As consumers often prefer to eat shellfish either raw or partially cooked, heat and expensive post-harvest treatments such as high-pressure processing are not always viable options for treatment. Therefore, regular microbiological assessment of shellfish growing waters or shellfish is conducted according to regional shellfish quality guidelines. Shellfish harvesting restrictions based on microbiological quality of shellfish growing waters or the shellfish themselves are used to mitigate risks, however, this places an economic burden on the shellfish industries. Norovirus outbreaks related to shellfish consumption remain regularly reported worldwide [6,7,8,9,10,11].

Norovirus is a major cause of viral gastroenteritis frequently associated with outbreaks in communal settings such as rest homes, hospitals and restaurants [6,12,13,14]. Each year norovirus is responsible for approximately 125 million foodborne cases worldwide [15,16], a proportion of which are associated with the consumption of norovirus contaminated shellfish. Accordingly, there is a need for rapid, cheap, reliable and user-friendly detection methods for the onsite/infield detection of norovirus in shellfish to identify the contamination points across the production line (i.e., farm to plate) to reduce public health risk and to minimize shellfish farm harvesting closures. This review evaluates the advantages and disadvantages of currently used methods for the detection of norovirus from shellfish. Furthermore, emerging technologies with the potential to detect norovirus from shellfish are assessed.

Due to the heterogeneous distribution of norovirus in shellfish and the requirement to process a minimum of 2 g of digestive tissue, at least 6–25 shellfish (depending on shellfish size) are used to recover norovirus from shellfish [17,18,19,20]. For the recovery of norovirus from shellfish tissue, different methods such as virus elution and concentration, and proteinase K digestion have been developed and evaluated [20,21,22,23,24,25,26,27,28,29,30,31,32]. Direct nucleic acid extraction from the digestive tissue of shellfish has also been used [33,34].

**Figure 1:**
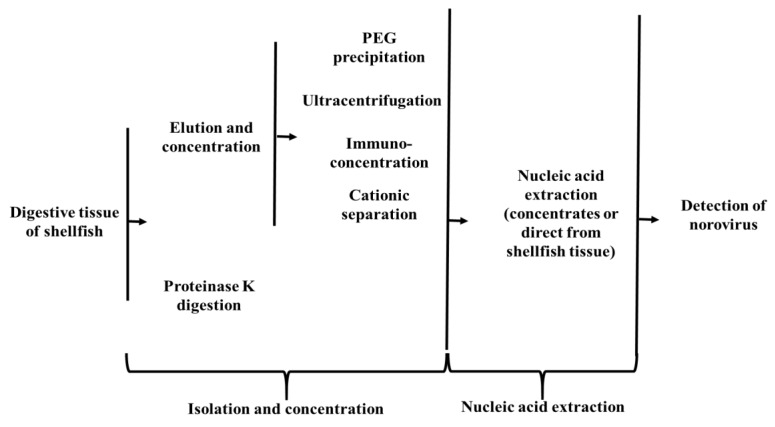
A flow diagram of current approaches to isolate, concentrate and detect norovirus from shellfish.

For the virus elution and concentration methods, recovery of norovirus (or viral surrogates) usually involves the release of viruses from the digestive tissue of shellfish using buffers (such as phosphate buffered saline, Tris-HCL buffer, alkaline glycine buffer) [20,21,24,30]. The eluted viruses can then be concentrated using polyethylene glycol (PEG) precipitation, ultrafiltration, ultracentrifugation, immuno-concentration or cationic separation (Figure 1). In some cases, the concentrated material is subjected to further clarification such as chloroform extraction, or additional ultracentrifugation.

The recovery rate of the elution and concentration approach has been reported to be highly variable (20–70 reverse transcription polymerase chain reaction unit (RT-PCR U)) within or between the protocols depending on the shellfish type and volume of shellfish digestive tissue [22,23,24,25,26,27,28,29,30] (Table 1). In addition, extraction processes can be time-consuming (requiring 1 to 3 days to process a batch of samples) [27]. An overview of the reported limit of detection (LOD) or recovery rate (%) of each method is presented in Table 1.

An alternative method to recover norovirus (and hepatitis A virus) from shellfish was developed that utilized the proteinase K enzyme to release the viruses from shellfish digestive tissue [22,35]. This method is simple, can be completed in a few hours and does not include a virus concentration step. The proteinase K digestion method has been extensively evaluated [20,30,31,32] and was included in the ISO 15216 standard method for the quantitative detection of norovirus and hepatitis A virus in 2017 [32]. The recovery rate of this method is reported to be higher (21 ± 15% to 34 ± 5%) than elution and concentration methods [20] (Table 1). An evaluation of the performance of the ISO 15216 method for the recovery and detection of norovirus and hepatitis A viruses in shellfish found that the limit of detection of norovirus (34 genome copies (GC)/g for norovirus GI and 53 GC/g for norovirus GII) was lower than for hepatitis A virus (198 GC/g) [36] (Table 1). 

Despite reducing the time for sample preparation, the proteinase K digestion method has the potential to inactivate norovirus during the recovery process as the enzyme activity relies on a 65 °C heat treatment [37]. Therefore, this approach may not be suitable for the downstream determination of virus infectivity, which is essential when trying to measure the potential public health risk associated with shellfish consumption. As such, further research should focus on improving the recovery efficiency of norovirus from shellfish with minimal impact on infectivity.

Several studies have demonstrated the successful application of direct nucleic acid extraction from shellfish digestive tissue using zirconium beads [33,34,38]. The method detection limit was reported to be 10 RT-PCR U from 0.15 g of oyster digestive tissue [34] (Table 1). Although direct nucleic acid extraction methods are promising, unless combined with another approach, they are not able to discriminate between infectious and non-infectious norovirus. As such, they are not ideal for evaluating potential public health risks associated with shellfish consumption. In addition, direct nucleic acid extraction may not successfully remove PCR inhibitors present in the shellfish tissues that can hinder the downstream detection.

## 2. Current Norovirus Detection from Shellfish

Reverse transcription quantitative PCR (RT-qPCR) is widely used for the detection and quantification of norovirus from shellfish [3,18,22,32,39,40,41]. Unlike conventional RT-PCR, RT-qPCR utilizes fluorescently labelled probes that allow for the simultaneous confirmation of the presence of the specific target. For norovirus, RT-qPCR methods are rapid, sensitive and specific but as virus quantification depends on the use of a calibration standard curve, variability in quantification can occur between laboratories. The detailed RT-qPCR protocol with a description of the suite of controls required for quantification was described in the ISO 15216 method [32].

Digital PCR (dPCR), with a RT step (RT-dPCR) for norovirus, can overcome the requirement for a standard curve [42,43]. dPCR is an absolute end-point nucleic acid quantitative technique based on dividing the sample into many thousands of partitions, analyzing each partition by PCR and using Poisson statistics, rather than an external calibration curve, to quantitate. One dPCR approach is droplet digital PCR (ddPCR). In ddPCR, oil nanodroplets in water are first prepared and then subjected to qPCR analysis. The number of positive and negative nanodroplets after the qPCR assay is calculated and as for conventional dPCR, Poisson statistics are used for quantification. Another advantage of dPCR/ddPCR over qPCR is that it is reported to give more accurate quantification and is less prone to inhibitors that may be present in nucleic acid extracted from shellfish, even after purification. For samples with a high concentration of target, quantification may not be possible without dilution. For instance, one study using ddPCR reported 100% saturation of positive droplets at 10^5^ target copies per µL template [44].

Loop-mediated isothermal amplification (LAMP), with a RT step for norovirus, is another molecular method that has the potential to detect norovirus from shellfish faster, cheaper and with equal sensitivity to RT-qPCR [45,46]. The LAMP method uses auto-cycling strand displacement DNA synthesis under isothermal conditions. While RT-qPCR/RT-dPCR uses expensive specialized equipment such as thermal cyclers, LAMP only requires a waterbath or heat block to maintain the isothermal conditions, with product measured using a turbidity, coloration or fluorescence-based detector. This means that RT-LAMP has the potential to be used on-site [45,46]. Fukuda and colleagues combined nucleic acid sequence-based amplification (NASBA) with a RT-LAMP assay (NASBA-RT-LAMP) and evaluated the sensitivity against RT-seminested PCR. The sensitivity of the NASBA-RT-LAMP assay for detecting norovirus in oysters was reported to be equivalent to the RT-semi-nested PCR, being able to frequently detect less than 100 genome copies of norovirus in oysters [46].

**Figure 2:**
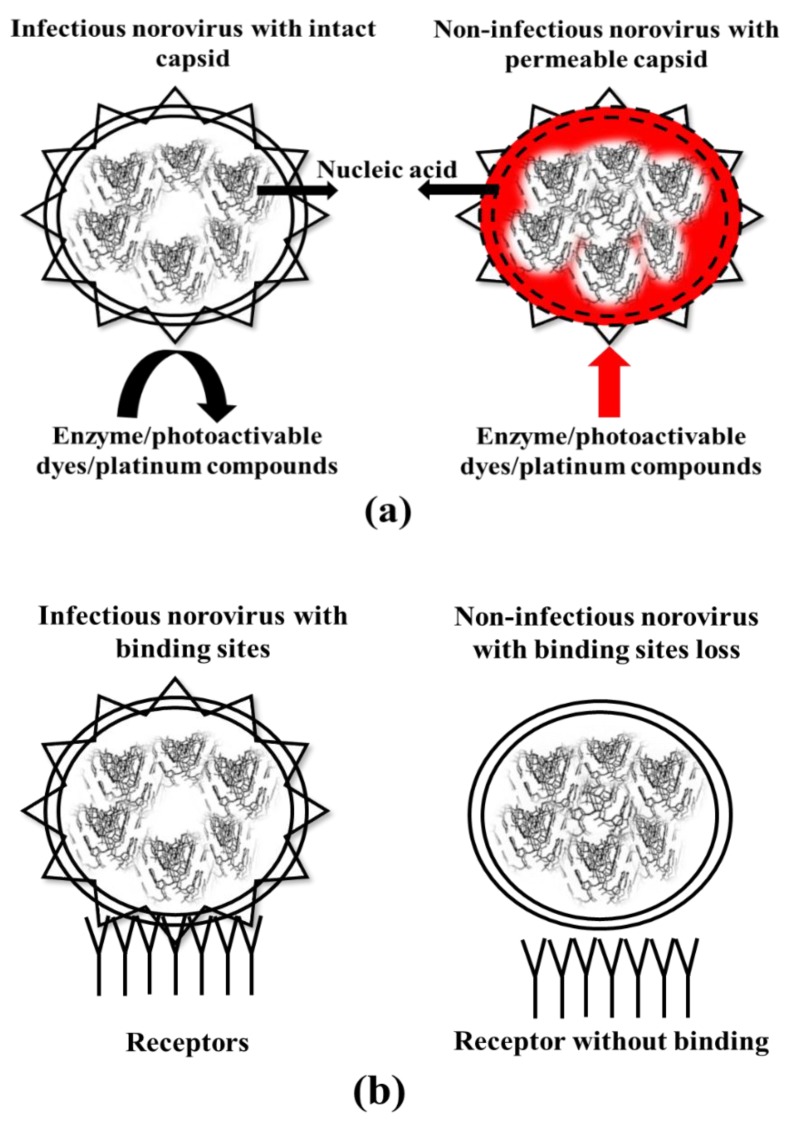
Mechanism of infectious RT-qPCR (**a**) and binding assays (**b**) for the differentiation between infectious and non-infectious norovirus. In the assay, enzymes or compounds enter the permeable capsid of non-infectious viruses and interact with the nucleic acid. The viral nucleic acid is then inactivated or degraded and hence cannot be detected in the subsequent qPCR assays. In the receptor binding assay, the binding site of the virus is lost on non-viable viruses allowing specific binding or capture of infectious viruses only.

Despite being rapid, sensitive and specific, molecular methods are unable to predict the infectivity of norovirus present in the shellfish. In recent years, RT-qPCR methods have been combined with a pretreatment such as enzymatic (RNase) [47,48,49,50,51], photoactivatable dyes (EMA, PMA, PMAxx and PEMAX) [52,53,54,55,56] and a platinum compound [57] for the selective detection of infectious norovirus. Other methods, such as porcine gastric mucin-binding [58,59,60,61] and in situ capture [62,63,64,65,66,67], have also been combined with RT-qPCR for this purpose. The working mechanism of modified RT-qPCR assays to determine infectivity is shown in Figure 2.

The applicability of these modified RT-qPCR assays is questionable due to their dependency on sample matrix, pathogen inactivation mechanism, treatment conditions, condition of binding site and lack of reproducibility [52,58,62,68]. As such, they may not provide an accurate estimation of infectivity. In addition, some studies have evaluated the applicability of these methods to determine the norovirus infectivity in shellfish and reported having limited success and inconsistent results [53,56]. These inconsistent results could be due to the use of different compounds or norovirus inactivation protocols such as heat, UV radiation and chlorination, which each damage norovirus by different mechanisms. For example, thermal inactivation can damage the norovirus capsid more effectively than UV radiation and chlorination. As a result, modified infectivity-based RT-qPCR assays may be more effective on thermally inactivated norovirus, and less applicable for UV radiation and chlorination treatments. 

## 3. Emerging Detection Technologies

Due to the limitations of current methods including costs, lengthy times to obtain results and the necessity of advanced laboratory equipment, infrastructure and skilled personnel, there is a need for rapid and easy detection techniques for norovirus from shellfish with minimal manual sample handling. Early and accurate onsite detection and identification of norovirus contamination in shellfish tissue will reduce costs by: (a) eliminating sample transportation, (b) holding products in cold storage while routine testing is conducted, (c) minimizing operating, infrastructure, equipment and personnel costs linked to testing laboratories, and (d) limiting the impact caused due to farm closures or product recalls. These early and onsite pathogen detection technologies would not only reduce the risk of foodborne illness but also provide greater product assurance. 

Recent advances in omics, nanotechnology, electrochemical and molecular detection technologies can improve the development of such rapid diagnostic devices [69,70,71,72,73,74,75]. In addition, advancements in 3D printing may improve the size and physical footprint of the detection devices and reduce their production cost [76]. 

### 3.1. Omics-Based Approaches

**Figure 3:**
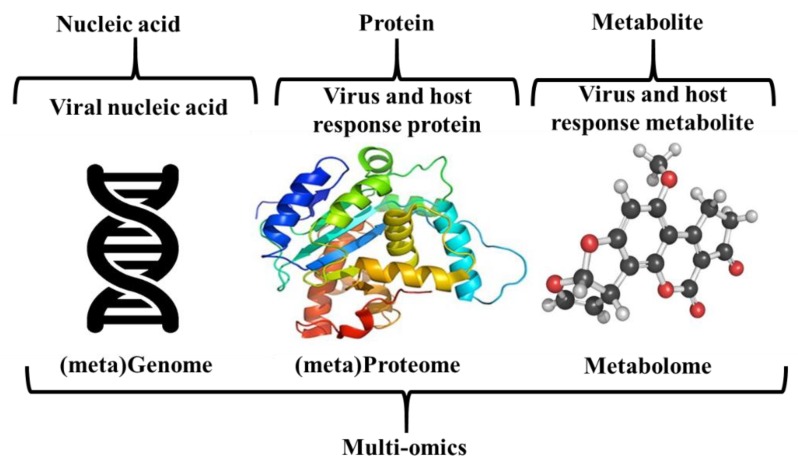
Omics-based virus monitoring approaches.

Omics-based approaches such as metagenomics, proteomics and metabolomics have the potential to deliver rapid diagnostic techniques towards food safety research [69]. The use of omics-based approaches could result in a paradigm shift for food safety testing, as seen for human and veterinary medicine where there have been developments in rapid and precise pathogen detection and characterization [70]. Figure 3 illustrates the omics platforms that have the most potential for monitoring and assessing the presence of norovirus in shellfish. 

#### 3.1.1. Metagenomics

Metagenomics is the analysis of total nucleic acids, including those from viruses that are present in a complex biological matrix. Viral metagenomics offers an alternative approach to a pathogen-specific molecular method [77]. Metagenomics involves high-throughput sequencing of RNA/DNA amplicons from a matrix, generating a large amount of genomic data. Bioinformatic analysis of the metagenomics datasets then identify and characterize the microbial communities which provides additional information regarding viral genomes present [78,79]. Because other human enteric viruses can also be present in shellfish [80], metagenomics has the potential to detect multiple pathogens from a single sample reaction. For example, metagenomics was used to identify multiple viruses from a oyster-related acute gastroenteritis outbreak in Osaka City in Japan, which were attributed to other pathogens such as astrovirus, sapovirus and rotavirus [80].

Despite a lack of published studies performing untargeted viromes sequencing from shellfish samples, metagenomics can be beneficial for public health and for shellfish safety. With the recent development in the MinION platform (Oxford Nanopore Technologies), metagenomics-based methods have the potential to be used in the field as necessary. Currently, viral metagenomics is expensive and requires skilled personnel to analyze the information to produce a confirmative result. In addition, the sensitivity and specificity of the metagenomics approach has not been fully evaluated and is in need of more research. 

#### 3.1.2. Proteomics

Metaproteomics is the analysis of proteins produced by an organism or population of organisms, and their expression in the presence of pathogens and viruses. The proteome is defined as the entire set of proteins that are, or can be, produced by a genome and is different among individuals, cell types, or even within the same cell at different times or growth phases. Proteomics encompasses the scientific research of the proteome, including protein composition, structure, levels and unique activity patterns [81,82]. In recent years, proteomics-based matrix-assisted laser desorption ionization time-of-flight mass spectrometry (MALDI-ToF MS) has emerged as a tool for pathogen identification and diagnostics from intact cells or cell extracts [81,83]. MALDI-ToF MS is a rapid and sensitive technique and has been used for food authentication [84] and the detection of foodborne and waterborne pathogens [85,86]. 

This technology primarily relies on the characterization of pathogens by analyzing the whole cell or viral proteome in a typical mass range *m/z* of 2–20 kDa [87]. One of the disadvantages of MALDI-ToF MS is that it is reliant on the existing spectral database of the mass fingerprints of the pathogen strains and is unable to identify new species of organisms. Proteomics can be complemented with other omics-based techniques such as metabolomics to develop a robust and reliable tool for pathogen identification and diagnostics. Similar approaches have been applied to assess food allergies and food safety in shellfish [88,89,90].

#### 3.1.3. Metabolomics

Metabolomics is the analysis of the small chemical compounds (metabolites, molecular weight <1.5 kDa) produced and consumed by an organism or a population of organisms because of their environmental and genetic potential (including exposure to viruses). The metabolome comprises the final downstream product of the genome, transcriptome, and proteome, which reflects the phenotype of a biological system [82]. Traditionally, metabolomics has been extensively applied in disease diagnosis [91], agriculture [92,93] and toxicology [94].

Research around ‘food metabolomics’ has gained momentum in the last decade. Several studies have applied this approach for the detection of foodborne pathogens (e.g., *Listeria* spp.) [95,96,97]. A considerable proportion of metabolomic-based applications are focused on food composition and traceability of foods, food quality and food safety [98,99,100]. For example, Aru et al. [101] and Alfaro et al. [102] applied a metabolomics approach to analyze changes in the metabolic profile of mussels under various food storage conditions, correlating observed metabolite signatures with microbial counts as potential biomarkers of spoilage. Nguyen et al. [103] utilized metabolomics to assess the tissue-specific immune response of New Zealand Greenshell^™^ mussel (*Perna canaliculus*) infected with *Vibrio coralliilyticus*. Nguyen et al. [103] concluded that such an approach could be used to rapidly assess infected mussels by assessing the mussel immune response to infection. Others have utilized metabolomics of shellfish to assess climate change impacts and environmental contaminants [104,105]. 

Although there is great potential for omics-based approaches to detect norovirus, these methods are in the early stages of development and have not been evaluated extensively for the detection of norovirus from shellfish. Extraction of nucleic acid, metabolites, and protein may hinder its onsite application. Limitations of omics-based approaches need to be considered so that public health risks are not overestimated. Despite the limitations, omics-based approaches have the potential to provide or identify biomarkers that can be used to develop rapid onsite diagnostic sensors or kits for norovirus detection.

### 3.2. Emerging Technologies for Onsite Detection

#### 3.2.1. Nanomaterials

Different nanomaterials, such as nanocrystals, quantum dots and graphene, are gaining interest as a potential agent for virus detection. For instance, functionalized rod-shaped, colloidal cellulose nanocrystals (CNCs) have been used for the detection of cowpea chlorotic mottle virus and norovirus virus-like particles (VLPs). Cationic polymer brush was generated on the surface of these CNCs to retain excellent dispensability and colloidal stability in water with the electrostatic binding of the VLP [106]. The capture of norovirus VLPs by modified CNCs was verified by size (dynamic light scattering measurement) and electron microscopy. 

Quantum dots (QDs) are small fluorescent labels made with CdSe-ZnS with unique emission properties on a single wavelength excitation. QDs were utilized on a Surface Plasmon Resonance (SPR) assisted immunoassay to detect norovirus VLPs [73]. Combining the SPR enhancement, intensity of auto-fluorescence, and excitation efficiency of quantum dots, the single-to-noise ratio was optimized to increase the sensitivity of the sandwich assay for the detection of norovirus VLPs from phosphate buffered saline (PBS). The newly developed assay was able to detect 100 VLPs from the PBS solution [73]. Another plasmonic sensor was developed by Junesch and colleagues, where the lipid bilayer membrane was developed for binding norovirus, enabling label-free and real-time detection [107]. The interaction of norovirus with glycosphingolipids induced negative membrane curvature or invaginations due to viral accumulation. This novel location specific sensor is superior to conventional SPR or to other planar detection surfaces and requires only an ordinary spectrophotometer in virus detection.

Novel nanostructure using a hybrid of graphene and gold is also being explored for the detection of norovirus VLPs [108]. Antibody conjugated graphene-gold nanoparticles catalyze the substrate to generate a visible blue color which is directly proportional to the concentration of the target. This nanostructure combined the enhanced Raman intensity and peroxidase-like catalytic activity of graphene and gold. This combinational approach allowed the assay to be 100 times more sensitive than conventional ELISA methods and detected 100 pg/mL of the target. 

Most of the nanostructures like nanoparticles are utilized in combination with Lateral Flow Assays (LFA) for onsite detection of target pathogens [75]. Traditional LFA has limited sensitivity due to the background signal and lower signal intensity of commonly-used gold or blue latex nanoparticles. Phase nanoparticles have been used as a reporter based on antibodies identified from sandwich ELISAs. Validation of the LFA was performed using both gold and phase nanoparticles and showed phage nanoparticle LFA had a100-fold lower LOD than the gold nanoparticle LFA using the same antibody pair. 

#### 3.2.2. Aptamer

**Figure 4:**
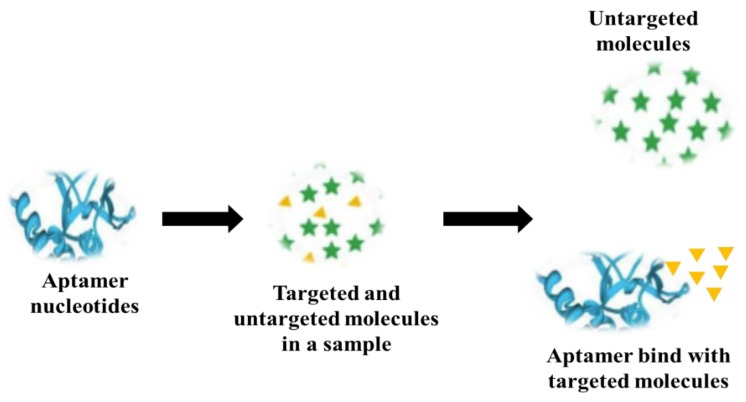
A flow diagram showing an aptamer-based norovirus detection approach.

Aptamers are short DNA, RNA and peptide-based sequences selected through systematic ligand evolution by an exponential enrichment (SELEX) process, which binds with the target (e.g., norovirus) based on its protein structure (Figure 4) [109,110,111]. Escudero-Abarca and colleagues developed four ssDNA aptamer candidates that targeted norovirus GII.4 but showed affinity to both GII.2 and GII.4 strains using an enzyme-linked aptamer sorbant assay [112]. The binding capacity of the aptamer was 13-14 VLPs, equivalent to that of a commercial anti-norovirus antibody (1 to 5 µg/mL). One of the four potential aptamer candidates (aptamer 25) developed by Escudero-Abarca and colleagues was coupled with a magnetic capture method for the detection of norovirus from artificially-contaminated lettuce. The capture efficiency of the magnetic capture method was 2.5 to 36% with a LOD of 10 RNA copies/lettuce sample [112]. 

To advance the aptamer-based detection technology, Moore and colleagues developed an aptamer-based technique not only to detect norovirus but also to demonstrate the confirmation-based binding. The aptamer was designed to target the P-domain protein of a norovirus GII.4 strain using *E. coli* to express and purify the P protein [109]. After SELEX, an aptamer named M6-2 was selected and confirmed for targeting norovirus GI.7, GII.2, GII.4 and GII.7 strains with low to moderate binding affinity. Magnetic particle-based capture and RT-PCR demonstrated a LOD of 4.88 log_10_ input genome copies (GC). These aptamers could also be used in combination with multiple sensing platforms for the detection of murine norovirus (used as a norovirus surrogate) or norovirus. One such example is the work by Wang and colleagues, where they combined aptamers with Micro-Electro-Mechanical Systems (MEMS) to develop a biosensor for norovirus [113]. 

#### 3.2.3. Biosensor-Based Detection

**Figure 5:**
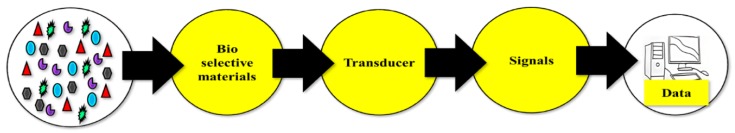
Sensor-based detection technologies for monitoring and assessing the presence of norovirus in shellfish.

A biosensor is an object that transduces biological signals to measurable optical, electrical or physical signals [114]. The output signals are either displayed, stored or analyzed to generate useful diagnostic information [115]. A biosensor generally possesses an antibody/antigen, enzyme, nucleic acid, phage, cell, or biomimetic membrane as a receptor or signal transducer. The most commonly used bioreceptors are antibodies, and nucleic acids such as aptamers. Figure 5 illustrates the mechanism of sensor-based detection technologies for monitoring and assessing the presence of norovirus in shellfish.

A miniaturized and portable MEMS-based electrochemical aptasensor was developed and evaluated for the detection of norovirus [113]. The electrode surface was functionalized with virus-specific fluorescent aptamers using drop-casting methods. The binding capability between the aptamer and the sensing electrode was evaluated by testing the sensor responses to different titers of murine norovirus. The MEMS aptasensor exhibited a rapid and clear response to different virus titers with a LOD of 50 plaque forming units (PFU)/mL.

Another electrochemical biosensor using Concanavalin A (ConA) conjugated with nanostructured gold electrode was developed to capture norovirus from food material within an hour with better specificity and sensitivity [116]. The study also demonstrated a LOD of norovirus from lettuce extract was 60 GC/mL with a specificity of 98% from a mixture containing hepatitis A virus, hepatitis E virus and norovirus. 

A label-free homogeneous assay was developed using a split G-quadruplex nano-tweezer to detect a partial norovirus RNA [117]. The nano-tweezer, with a single signal-transducing molecule, could self-assemble from three single-stranded DNA molecules by simple mixing. Upon recognition of norovirus RNA, the signal molecule structure changed and restored its activity hence producing the detectable signal. The LOD was reported at 4 nM. 

Multiple other biosensor assemblies have been published for potential norovirus detection from various sample sources [73,107,118]. For example, a thioglycolic-capped CdZnSeS quantum dot probe was developed for the detection of norovirus RNA with a high photo-luminescence quantum [118]. The sensitivity of this technology was reported to be 8.2 viral copies/mL with a specificity of 98%. Similarly, a gold-immobilized cysteine-incorporated peptide-based electrochemical biosensor was also evaluated for the detection of norovirus [119]. The reported LOD of this method was 7.8 copies/mL PBS. The described proof-of-concept studies showed potential for application to miniaturized micro-devices as a diagnostic tool for onsite detection of norovirus or other enteric viruses from shellfish.

#### 3.2.4. Microfluidic Technology 

**Figure 6:**
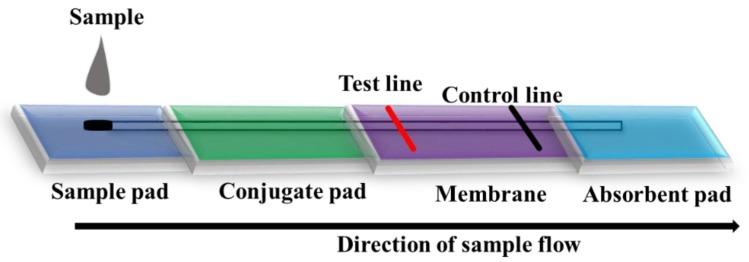
Flow diagram of the lateral flow assay (LFA) detection method.

Microfluidic technology is the miniaturization of molecular assays, which improves analytical performance by decreasing the consumption of reagents, detection time and human errors; while increasing sensitivity, reliability and the ability to detect multiple species of pathogens by integrating all necessary steps onto a single handheld disposable device [115,120]. Based on these advantages, microfluidic techniques offer promise for the rapid detection of norovirus from shellfish. During the assay, the sample passes through different regions on the microfluidics device either by capillary action or by pressure-driven by pumps. Multiple reactions of virus capture, isolation and identification can occur in different sections of microfluidic devices inside a closed system. Assays in closed systems ensures automated control of all steps and can reduce human errors and increase accuracy, reproducibility, and reliability of test results (Figure 6). Currently, there are two types of microfluidic technologies available for the detection of norovirus, (a) micro total analysis systems (lab-on-a-chip (LOC)) and, (b) paper-based analytical systems. 

Typically, the LOC system is a silicon, glass, and polymer-based chip where all of the processes for detection can be completed and results can be obtained within a short period of time [75,121,122,123]. In addition to the advantages of conventional microfluidics, such as size, speed and reduced sample amount, paper-based LOC also adds an inexpensive multiplexed setting [124]. Paper is considerably easy to source, cheap, biodegradable and, most importantly, easy to modify chemically. Other advantages of paper devices include requiring no external power sources, a high ratio of surface-to-volume and minimal technical expertise requirements. Paper-based microfluidics, compared to microfluidics with LOC formats, has significant advantages such as it is a simpler technology and has reduced costs. However, paper-based microfluidics has issues in sample retention and evaporation that makes it less suitable for the detection of a low concentration of pathogens in a particular sample. 

LOC modules have been used to detect murine norovirus with drop-based microfluidics [125]. A microfluidic platform combined with RT-dPCR was developed to amplify, detect and characterize genetic recombination between two murine norovirus strains. Another LOC-based norovirus detection method incorporated micro-bead beating to capture the virus and later this was used to lyse the virus inside the closed system [123]. This was achieved by switching the surface charge of the nanoparticles. An isothermal RNA amplification method and NASBA was utilized to detect murine norovirus from artificially contaminated oysters within 4 h. Viral RNA amplification and subsequent detection was achieved with LOD of 100 PFU of murine norovirus per oyster. Another example of the application of LOC for norovirus detection in environmental and food samples is the use of microfluidic RT-qPCR. A microfluidic RT-qPCR has already been used for the simultaneous quantification of eleven major human viruses including enterovirus, Aichi virus, adenovirus, astrovirus, sapovirus, rotavirus, norovirus, hepatitis A virus and hepatitis E virus from environmental water samples [126]. High throughput quantitative information can be obtained with detection limits of 2 GC/μL of DNA or cDNA. The recent advancement on omics-based technology, nanomaterials, nanoenzymes, aptamers and biosensors could be utilized to develop more sensitive, cheap and rapid microfluidic devices in the near future. 

In this review, we evaluated the advantages and disadvantages of the current detection methods and emerging technologies for the detection of norovirus from shellfish. Current nucleic acid-based detection methods including RT-qPCR, RT-dPCR and RT-LAMP/NASBA-RT-LAMP methods are rapid and sensitive and can be cheap (LAMP). However, these methods are unable to provide information on the infectivity of norovirus in shellfish. Molecular methods have beenmodified to inform on the infectivity of norovirus but still have limitations. In 2016, an in vitro cultivation method for norovirus using human intestinal enteroids was reported [127]. While the method is time consuming, expensive and still being optimized, the ability to culture norovirus will provide valuable infectivity information [128] and will enable better assessment of current detection methods to selectively detect infectious norovirus. Due to the limitations of current methods including cost, lengthy times to obtain results and the necessity of advanced laboratory equipment, infrastructure and skilled personnel, there is a need for rapid and easy detection techniques for norovirus from shellfish with minimal manual sample handling. Different rapid detection methods including aptamers, biosensors and microfluidic devices have been developed and evaluated for the rapid and sensitive detection of norovirus. So far, all emerging detection technologies have been tested using a simple matrix with a known concentration of norovirus. However, shellfish is a complex food matrix containing polysaccharides, glycogen and other compounds that may affect the efficacy of these emerging technologies. More research will be required to evaluate the performance of these new technologies in complex sample matrices such as shellfish. In addition, the continuous evolution of the norovirus RNA genome is another challenge that needs to be considered when developing onsite detection kits using emerging technologies. Despite the limitations, these technologies have the potential to be rapid and user-friendly detection kits that can be used for the detection of norovirus in real time. 

## Figures and Tables

**Table 1:** Limit of detection or recovery rates (%) of viruses from shellfish using various methods.

Methods	Concentration Method (Where Applicable)	Shellfish (Weight)	Viruses	Limit of Detection/Recovery Rate	References
Elution and concentration	PEG	Oysters (25 g)	Norovirus	22 RT-PCR U	[21]
		Mussels (2 g)	Norovirus GII	20 RT-PCR U	[24]
		Mussels (2 g)	Rotavirus	10 RT-PCR U	[25]
		Oysters (4 g)	Rotavirus	1.39 GC/4 g	[27]
		Mussels (4 g)	Rotavirus	1.39 GC/4 g	[27]
		Cockles (4 g)	Rotavirus	1.39 GC/4 g	[27]
		Mussels (1.5 g)	Mengovirus	1.8%	[28]
		Oysters (1.5 g)	Mengovirus	1.2%	[28]
		Oysters (1.5 g)	Norovirus GI	70 RT PCR U/g	[3]
		Oysters (1.5 g)	Norovirus GII	70 RT PCR U/g	[3]
	Ultracentrifuge	Oysters (25 g)	Hepatitis A virus	9.9%	[23]
	Cationic separation	Oysters (5 g)	Hepatitis A virus	20 RT-PCR U	[26]
Proteinases K digestion	Not applicable	Oysters (1.5 g)	Norovirus GI	20.5 ± 14.7%	[20]
		Oysters (1.5 g)	Norovirus GII	33.6 ± 5.3%	[20]
		Mussels (25 g)	Norovirus GI	3%	[30]
		Mussels (25 g)	Norovirus GII	3.5%	[30]
		Oysters (3 g)	Norovirus GI	34 GC/g	[36]
		Oysters (3 g)	Norovirus GII	53 GC/g	[36]
		Oysters (3 g)	Hepatitis A virus	198 GC/g	[36]
Direct RNA extraction	Not applicable	Oysters (0.15 g)	Norovirus	10 RT-PCR U	[34]
		Oysters (5–50 g)	Hepatitis A virus	8 PFU	[38]

PEG, polyethylene glycol; PFU, plaque forming units; RT-PCR U, reverse transcription polymerase chain reaction unit; GC, genome copies; GI, genogroup I; GII, genogroup II.
